# The Impact of the COVID-19 Pandemic on the Neck of the Femur and Hip Fracture Surgery Volumes in the Lazio Region

**DOI:** 10.3390/ijerph19084704

**Published:** 2022-04-13

**Authors:** Francesca Mataloni, Luigi Pinnarelli, Paola Colais, Jacopo Savastano, Danilo Fusco, Marina Davoli

**Affiliations:** Department of Epidemiology, Lazio Regional Health Service, 00147 Rome, Italy; f.mataloni@deplazio.it (F.M.); l.pinnarelli@deplazio.it (L.P.); j.savastano@deplazio.it (J.S.); d.fusco@deplazio.it (D.F.); m.davoli@deplazio.it (M.D.)

**Keywords:** COVID-19, surgery, fracture, hip, neck of the femur, hospitals, orthopedics

## Abstract

This study compares surgery volumes for fractures of the neck of the femur (FNF) and hip replacements during the COVID-19 pandemic compared with previous years. Historical (2018–2019) and pandemic (2020–2021) surgery rates for FNF and hip replacement in Lazio, adjusted for age and gender, were calculated per period and compared with a Poisson regression model. For hip replacement surgery, a comparison of different types of hospitals was also made. Before COVID-19’s spread, no difference was found in the volume of surgery of both interventions. From the lockdown to the end of 2021, a decrease in surgery volumes for FNF with stabilization between summer 2020 and summer 2021, as well as an additional decline beginning at the start of Omicron’s spread, were found. Hip replacement surgeries showed a greater decline during the lockdown period and increased during summer 2020 and during the Delta wave period. The increment in hip replacements, mainly observed in 2021, is due to private and religious hospitals. These results highlight that the pandemic emergency, caused by SARS-CoV-2, has had an important indirect effect on the population’s health assistance in the field of orthopedics.

## 1. Introduction

The COVID-19 pandemic has caused direct and indirect consequences to the population’s health. More than 460 million people have been infected to date, with more than 6 million confirmed deaths worldwide [1]. From March 2020 to date, in Italy, more than 13 million people have been infected, causing more than 157,000 deaths. The pandemic has also caused an indirect effect on the population’s health, particularly due to the government’s request for people to “stay at home,” the fear of being infected, and the lack of hospital beds. Several studies have analyzed the indirect impact of COVID-19 on treatment and hospital care [2,3,4,5,6]. A recent study conducted in Italy analyzed indicators of volume and performance in three clinical areas: cardiology, oncology, and orthopedics, and found a decreased volume of hospitalizations during the first seven months of 2020 compared to previous years [2]. Despite the decrease in the volume of hospitalizations during 2020, a reduction in the proportion of timely interventions was not observed. A systematic review from January 2020 to 25 February 2021 showed that the clinical care of patients with acute cardiovascular conditions, their management, and outcomes were adversely impacted by the COVID-19 pandemic [3]. Indirect effects of the pandemic were also found in primary care in the UK [4]; in fact, this study showed a considerable reduction in primary care contacts, particularly for diabetic emergencies, depression, and self-harm, after the introduction of population-wide restrictions. Several studies analyzed the indirect impact of the pandemic on Orthopedic Trauma [5,6,7,8,9,10,11,12]. Studies conducted in 2020, at the beginning of the pandemic, found higher rates of inpatient mortality in hip fracture patients with concomitant COVID-19 infection than those without concomitant COVID-19 infection, along with increased morbidity and mortality in patients with hip fractures complicated by COVID-19 [7,8]. A recent study conducted in California analyzed hip fracture surgery volumes in 2020 and 2021 by periods compared to previous years [9]. They found a significant decrease in intervention rates from March 2020 to the end of the summer, and also between November 2020 and the end of February 2021.

Both hip fracture injuries and fractures of the neck of the femur account for 100,000 hospitalizations per year in Italy and particularly affect the older population [13]. Several studies have demonstrated the importance of timely intervention in reducing adverse events in patients hospitalized for hip fractures [9,14,15]. In Italy, both surgeries are offered free of charge for all citizens through direct reimbursement of the national health service (NHS) to both private and public hospitals. Orthopedic surgery for hip fracture injuries is mainly managed by public hospitals, compared to hip replacement, which is mainly performed by private hospitals. The case of Lazio is particularly interesting for analyzing the impact of the pandemic on the volume and organization of orthopedic surgery because of the different types of hospitals reimbursed by the NHS: public, private, and religious. What characterizes religious hospitals is their ownership; they do not belong to the public health service but instead belong to religious orders. A religious hospital has the structural requirements and the organization of services that must be qualitatively equal to public structures. During the pandemic, all of these types of hospitals were directly and indirectly involved in the different phases of the pandemic, either through offering COVID-19 care or through the delocalization of elective surgery.

This study compares the volumes of surgery for fractures of the neck of the femur (FNF) and hip replacement during the COVID-19 pandemic compared to previous years and analyzes the impact of the organization of care across the different types of hospitals.

## 2. Materials and Methods

Our data were extracted from the Hospital Information System (HIS) database, which collects all hospital admissions of Lazio, including both clinical information (ICD 9 CM code of diagnoses and procedures, dates of admission and discharge, and status at discharge) and demographic information (age, gender).

We considered all patients discharged from any hospital in Lazio between 1 January 2018 and 31 December 2021 who had FNF surgery or hip replacement.

For FNF, we considered all patients diagnosed with fracture of the neck of the femur (ICD-9 cm diagnosis codes 820.0–820.9) and femur fracture surgeries. The surgeries were identified by the following ICD-9-CM codes:

- Total or partial replacement (81.51, 81.52);
- Reduction in fracture (79.00, 79.05, 79.10, 79.15, 79.20, 79.25, 79.30, 79.35, 79.40, 79.45, 79.50, 79.55);

For hip replacement surgery, we considered all patients admitted to hospitals in Lazio with the following codes of procedure (ICD9-CM procedure codes 81.51, 81.52, 81.53, 00.70, 00.71, 00.72, 00.73, 00.85, 00.86, 00.87).

To evaluate differences in surgery volumes during the different phases of the pandemic, eight periods were defined:

1. Pre-COVID-19 surge (1 January 2020–8 March 2020);
2. Italian lockdown and first surge (9 March 2020–31 May 2020);
3. Summer 2020, post-lockdown (1 June 2020–31 August 2020);
4. Fall 2020, second surge (1 September 2020–31 December 2020);
5. Early vaccination phase (1 January 2021–30 April 2021);
6. Late vaccination phase (1 May 2021–31 August 2021);
7. Delta wave (1 September 2021–31 October 2021);
8. Start of the Omicron spread (1 November 2021–31 December 2021).

Surgeries observed during the pre-pandemic years were defined as the mean of surgeries observed in 2018 and 2019 in each defined period.

For the two populations (FNF surgery and hip replacement), both historical and pandemic surgery rates, adjusted for age and gender, were calculated by period and compared using rate ratios (RRs) with a Poisson regression model.

Furthermore, a comparison between the periods for different types of hospitals was also made for hip replacement surgery. Four categories of the hospital were defined: public hospital corporations and teaching hospitals, local health unit hospitals, religious hospitals, and private hospitals. Adjusted surgery rates and rate ratios were calculated for each type of hospital and period.

Statistical analysis was performed using SAS Enterprise guide version 7.1, and R version 4.0.3.

## 3. Results

The number of patients admitted to the hospital for FNF surgery in 2020–2021 was 16,102, compared to 17,425 in 2018–2019. This study shows that this difference was not equally distributed in time, and the pandemic phases influenced the number of admissions and the incidence (Table 1). In particular, we did not observe relevant differences in the pre-COVID-19 period in terms of the volume of FNF surgery between 2020 and 2018–2019, while in the following periods, these differences started to rise. Similarly, in the first surge period, corresponding to the Italian lockdown, we observed a significant decrease in the surgery rate (1.50 vs. 1.86 cases per 1000 person-days; RR 0.81, 95% CI 0.76–0.86). During the following periods, the differences in the surgery rates were lower than the lockdown ones but were still significant, except for the last period with the beginning of the Omicron spread, where we saw the largest difference (1.47 vs. 1.93 cases per 1000 person-days; RR 0.76, 95% CI 0.71–0.82).

In 2020–2021, we found 18,102 patients with hip replacement surgery compared to 19,148 in 2018–2019 (Table 2). After the pre-surge period, where the surgery rate in 2020 was comparable to 2018–2019, the RR showed an important reduction during the second period (1.23 vs. 2.21 cases per 1000 person-days; RR 0.56 95% CI, 0.52–0.59). In the post-lockdown period, the surgery rate of hip replacement in 2020 was similar to 2018–2019 (1.68 vs. 1.67 cases per 1000 person-days; RR 1.01, 95% CI 0.95–1.07). The following COVID-19 periods (2020–2021) were characterized by lower surgery rates compared to 2018–2019, until the Delta wave, where surgery rates for hip replacements were significantly higher compared to the historical period (2.25 vs. 2.01 cases per 1000 person-days; RR 1.12, 95% CI 1.05–1.20).

**Figure 1:**
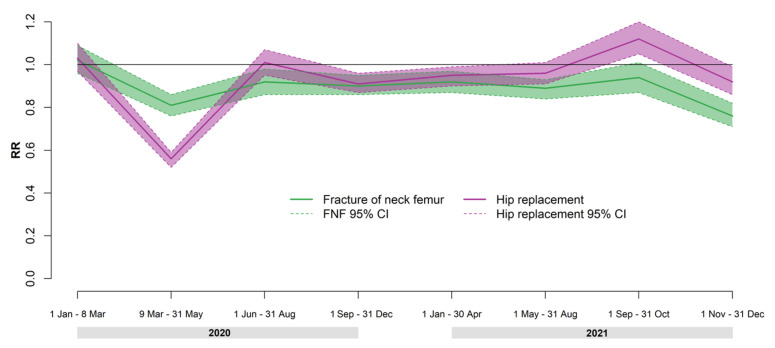
Rate ratios (RRs) and 95% confidence intervals (95% CIs) of fractures of the neck of the femur and hip replacement surgeries by period in 2020–2021 compared to 2018–2019.

Figure 1 reports the rate ratios of surgery rates for FNF and hip replacement according to time period.

Relative ratios of hip replacements showed a similar trend to FNF by period but with wider oscillations. The decrease in surgery rates during the lockdown period and the increase observed during the Delta wave period were greater for hip replacement surgery rates than FNF surgery rates.

**Figure 2:**
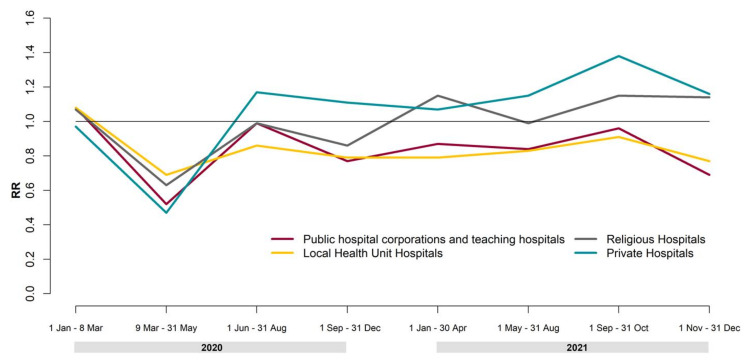
Rate ratios (RRs) of hip replacement surgeries by period and type of hospital in 2020–2021 compared to 2018–2019.

Figure 2 reports the results of the analysis of hip replacement surgery by period and the type of hospital. As we have already seen, in the 2020 lockdown period, we observed a relevant decrease in hip replacement surgery, and this drop affected all kinds of hospitals. Starting from the post-lockdown period, we observed a fast increase in hospitalizations in private hospitals (RR = 1.17, 95% CI 1.05–1.29). On the other hand, public hospital corporations, teaching hospitals, and local health unit hospitals showed a reduction in their hospitalizations in 2018–2019. In 2020, the trend of religious hospitals was very similar to that of public structures; in 2021, we observed an increase in surgical activity comparable to that of private hospitals.

## 4. Discussion

This study aimed to evaluate the effect of the COVID-19 pandemic on the volumes of surgery for fractures of the neck of the femur (FNF) and hip replacement and to analyze the impact of different organizations of care across the different types of hospitals in Lazio. An overall decrease in FNF and hip replacement surgery volumes in 2020–2021 was found compared to the previous two-year period (2018–2019). During the first period, before COVID-19’s spread, no difference was found in the volume of surgery of both interventions. From the lockdown (March 2020) to the end of 2021, we found a remarkable decrease in surgery volumes for FNF. The highest decrease was observed in the first period of full lockdown in all of Italy, which corresponded to the beginning of the pandemic surge in the north of Italy; in the rest of the peninsula, including Lazio, the number of infections was still low. Then, we observed a stabilization until the last period of 2021, characterized by the start of the Omicron spread. Hip replacement surgery showed a more variable trend, with a greater decline during the lockdown period and an increase in volumes comparable to 2018–2019 during summer 2020. Afterward, we observed a further decline in the fall of 2020, when the second surge also hit the region (a maximum of about 2800 new daily cases compared with 222 in the first surge) [16]; from fall 2020 to summer 2021 (the late vaccination phase), surgery volumes remain stable to a lower level compared to 2018–2019, and then increased again during the Delta wave period (September–October 2021), reaching a higher number of surgeries. During the last period of 2021, with the start of the Omicron spread, surgeries for hip replacement decreased again. The analysis of hip replacement specific to the types of hospitals shows that the increment of activity, observed mainly in 2021, is due to private and religious hospitals; moreover, private hospitals increased their surgical activity between summer 2020 and the end of 2021 compared to previous years. In fact, the volume of hip replacement surgeries in private hospitals was about 37% in 2018–2019, while in 2021, this percentage increased to almost 44% (data not shown). This phenomenon was not observed in FNF surgeries, which is mainly an unplanned activity managed by public hospitals. Thus, the differences observed depend on the type of hospital ownership. In particular, private hospitals and hospitals owned by religious orders increased their planned activity, while public hospitals had to manage emergency access. Ultimately, the largest increase in planned activity was observed in private hospitals reimbursed based on actual hospitalizations, while the smallest increase was observed in public hospitals reimbursed on the basis of a predetermined budget.

The decline in FNF surgeries from the lockdown of 2020 to the end of 2021 mirrored a decrease in hip fractures themselves, and this could be explained by a decreased risk of falls attributable to a strong reduction in outdoor activity or by a greater presence of family members at home in the case of elderly patients, although these results deserve more research [9]. Regarding hip replacement, the huge decline in intervention during the lockdown period could be explained by the main characteristics of this intervention that, differently from the FNF, it is generally planned and not an emergency. The variable trend, observed from summer 2020 to the end of 2021, could depend on the alternation of periods in which planned surgery decreased due to the worsening of the pandemic and regional indications to reduce scheduled activities [17], along with periods in which the pressure of the pandemic on hospitals decreased and planned surgeries were rescheduled.

The decline in orthopedic surgeries observed in Lazio (central Italy) during the COVID-19 pandemic was also reported by studies conducted in other countries [6,9,10,18,19]. In the USA, Zhong et al. showed that the COVID-19 pandemic significantly impacted not only the volume of hip fractures (16,068 hip fractures were observed in 2019 compared with 7498 in 2020) but also other outcomes such as the length of hospital stay or complications [10]. Okike et al. analyzed data on hip fracture surgeries in a California hospital by periods [9]. Their analysis confirmed the decline in surgery in 2020, but, differently from our results, they found no significant reduction in 2021 compared to previous years, except for in winter. Additionally, in England, Wales, and Northern Ireland, a decline in orthopedic surgery activities was reported [18]; moreover, they found a stable proportion of timely intervention but an increase in 30-day mortality rates in March 2020 for hip fracture patients. A study conducted in Romania that analyzed data from three clinics reported a decline in activity in April 2020 in the regional trauma center (−23.8%) and halted activities in the other two clinics [6]. A previous Italian study analyzed data of femur fracture surgeries in 10 Italian orthopedic centers from 23 February 2020 to 3 May 2020 [19]. The 10 Italian centers involved in the study included hospitals in the north, center, and south of the country. In the period analyzed, they found a decrease in femur surgeries in northern and southern hospitals and no differences in the two hospitals in Lazio. Our study provides a more complete picture because it does not restrict the analysis to a few centers but analyses data from all hospitals in the region throughout a longer time period. At the same time, our results are not generalizable to all of Italy because the impact of the pandemic has not been homogeneous throughout Italy due to the different rates of infection of COVID-19 in regions and for the capacity of the Regional Health Services to contain the health emergency.

Our study is based on administrative data, so it is not possible to evaluate the quality and completeness of the data, which could present some biases, such as missing data, underreported codes, or errors. In spite of this, the use of data from HIS allowed us to analyze data from all hospitals in the region, providing data that were similar to those from more specialized databases [20].

These results highlight that the pandemic health emergency, caused by SARS-CoV-2, has had an important, indirect effect on the population’s health assistance in the field of orthopedics. For this reason, tools aimed at decision support are necessary, mainly for pathologies, such as fractures of the neck of the femur, that require timely intervention [14,15,21]. Furthermore, we observed that the resilience of private hospitals was greater than the public counterpart. This is presumably attributable to the greater involvement of public structures in managing the COVID-19 pandemic, even in the subsequent epidemic phases [22]. The region adopted a flexible plan organized in different phases during which full hospitals or specific wards were converted into COVID hospitals. According to the different phases, planned, non-urgent surgical activities were suspended and reactivated following the different pandemic surges. Moreover, private hospitals were asked to supply hospital beds to the Regional Health Service. On the other hand, as reported in other studies [23], the differences in the type of reimbursement between private and public health structures may also have played an important role in the provision of health services.

## 5. Conclusions

The results of this study underline the predictable reduction in surgeries due to the first COVID-19 lockdown for both hip replacement and FNF. Furthermore, we saw that the recovery after the first wave differed for the two types of surgeries. Hip replacement surgery returned to an activity level comparable with the historical period (2018–2019), while FNF surgery remained slightly under its previous level. Evidently, the pandemic period has had a big impact on the organization of the public health system, and orthopedic fields were not protected from the subsequent restrictions. In contrast, we observed a bigger recovery in private hospitals’ hip fracture surgery volume. The comparison of surgery volumes during the SARS-CoV-2 epidemic with previous periods can provide useful information for the promotion and improvement of the planning and management of critical situations, such as that caused by a new infectious agent [24]; furthermore, monitoring health assistance to the public is a useful tool for future reorganization of the healthcare system.

## Figures and Tables

**Table 1:** Surgery for fracture of the neck of the femur (FNF) during COVID-19 pandemic (2020–2021) compared with historical time periods (2018–2019), adjusted surgery rates 1000, and ratios (RRs).

Period		FNF Surgery		Adjusted Surgery Rate *1000		RR	95%CI
		2020–2021	2018–2019	2020–2021	2018–2019		
Pre-COVID-19 surge	1 January–8 March	1784	1698	1.96	1.92	1.02	0.96–1.09
Italian lockdown and first surge	9 March–31 May	1690	2037	1.50	1.86	0.81	0.76–0.86
Summer 2020, post-lockdown	1 June–31 August	1938	2060	1.57	1.72	0.92	0.86–0.98
Fall 2020, second surge	1 September–31 December	2704	2918	1.66	1.83	0.90	0.86–0.95
Early vaccination phase	1 January–30 April	2824	2966	1.74	1.90	0.92	0.87–0.97
Late vaccination phase	1 May–31 August	2605	2830	1.56	1.76	0.89	0.84–0.93
Delta wave	1 September–31 October	1343	1382	1.63	1.74	0.94	0.87–1.01
Start of the omicron spread	1 November–31 December	1214	1536	1.47	1.93	0.76	0.71–0.82

**Table 2:** Surgeries for hip replacement during COVID-19 pandemic (2020–2021) compared with historical time periods (2018–2019), adjusted surgery rates *1000, and rate ratios (RRs).

Period		Hip Replacement Surgery		Adjusted Surgery Rate *1000		RR	95%CI
		2020–2021	2018–2019	2020–2021	2018–2019		
Pre-COVID-19 surge	1 January–8 March	1925	1822	2.12	2.05	1.03	0.97–1.10
Italian lockdown and first surge	9 March–31 May	1379	2428	1.23	2.21	0.56	0.52–0.59
Summer 2020, post-lockdown	1 June–31 August	2066	2004	1.68	1.67	1.01	0.95–1.07
Fall 2020, second surge	1 September–31 December	3090	3318	1.90	2.08	0.91	0.87–0.96
Early vaccination phase	1 January–30 April	3275	3349	2.02	2.14	0.95	0.90–0.99
Late vaccination phase	1 May–31 August	2882	2906	1.74	1.81	0.96	0.91–1.01
Delta wave	1 September–31 October	1848	1602	2.25	2.01	1.12	1.05–1.20
Start of the omicron spread	1 November–31 December	1637	1716	1.99	2.15	0.92	0.86–0.99

## Data Availability

The data are available from Lazio Region with its permission and upon reasonable request, by contacting direttore.direzionesalute@regione.lazio.it.
